# Effect of the therapeutic modulation of APT20TTMG on U1 snRNP complex assembly in Alzheimer’s disease

**DOI:** 10.1002/alz.089478

**Published:** 2025-01-09

**Authors:** Rafael Bottos, Camila Zimmer, Vanessa Sinatti, Ericks Soares, Michael Rafii, Luc Buee, Caio Leal

**Affiliations:** ^1^ Aptah Bio Inc., San Carlos, CA USA; ^2^ Vesper Biotechnologies, Dover, DE USA; ^3^ University of Southern California, San Diego, CA USA; ^4^ Université de Lille, Lille, Hauts‐de‐France France

## Abstract

**Background:**

Alzheimer’s disease (AD) is the most common cause of dementia worldwide. It is characterized by dysfunction in the U1 small nuclear ribonucleoproteins (snRNPs) complex, which may precede TAU aggregation, enhancing premature polyadenylation, spliceosome dysfunction, and causing cell cycle reentry and death. Thus, we evaluated the effects of a synthetic single‐stranded cDNA, called APT20TTMG, in induced pluripotent stem cells (iPSC) derived neurons from healthy and AD donors and in the Senescence Accelerated Mouse‐Prone 8 (SAMP8) model.

**Method:**

Following a 7‐day treatment with 5 concentrations of APT20TTMG in iPSC‐derived healthy and AD neurons, mitochondrial activity, glutamate release, and TAU levels were analyzed. The most effective concentration was chosen for the analysis of RNA‐seq differential expression and 3’ untranslated region and coding sequence (UTR/CDS) expression ratios for U1‐70K, SNRP200, MAPT, and APP genes. Levels of U1‐70K, TAU, Aβ, and GFAP were analyzed in 20‐week‐old female SAMP8 mice (Animal Welfare Act, Austria: BGBl. II Nr. 542/2020, BGBl. I Nr. 76/2020, BGBl. I Nr. 86/2018, BGBl. I Nr. 8/2022), treated with 0.3 µg APT20TTMG infused continuously for 42 days (i.c.v.).

**Result:**

In addition to unaffected mitochondrial activity and glutamate release, treatment decreased TAU in AD neurons, with no changes in healthy neurons. Metabolic pathway enrichment and network analysis identified four prominent clusters containing differentially expressed genes (DEGs) associated with pathways like cell cycle, PI3K‐Akt signaling pathway, fatty acid metabolism, MAPK signaling pathway, and steroid biosynthesis. APT20TTMG also corrected target assembly, avoiding premature polyadenylation of all analyzed genes. In vivo, treatment decreased U1‐70K in both cortex and hippocampus, as well as Aβ in cortex, and insoluble pTau in hippocampus. It also reduced gliosis (GFAP) in the cortex of animals.

**Conclusion:**

APT20TTMG specifically decreases TAU protein in AD neurons, with a wide safe effect for human brain cells. Its ability to correct the U1 snRNPs dysfunction prevents premature polyadenylation in key genes and leads to a favorable enrichment of DEGs related to pathways deregulated in AD. It also decreases important hallmarks of the disease, in addition to controlling severe astrogliosis. These data evidence the great potential of utilizing APT20TTMG as a disruptive approach for neuronal protection in AD.

